# Intravenous S-Ketamine Does Not Inhibit Alveolar Fluid Clearance in a Septic Rat Model

**DOI:** 10.1371/journal.pone.0112622

**Published:** 2014-11-11

**Authors:** Christian Fastner, Heimo Mairbäurl, Nina C. Weber, Koen van der Sluijs, Florian Hackl, Lorenz Hotz, Albert Dahan, Markus W. Hollmann, Marc M. Berger

**Affiliations:** 1 Department of Anesthesiology, University Hospital, Heidelberg, Germany; 2 Medical Clinic VII, Sports Medicine, University Hospital, Heidelberg, Germany; 3 Laboratory of Experimental Intensive Care and Anesthesiology, University of Amsterdam (AMC), Amsterdam, The Netherlands; 4 Department of Anesthesiology, University Medical Center, Leiden, The Netherlands; 5 Department of Anesthesiology, University of Amsterdam (AMC), Amsterdam, The Netherlands; 6 Department of Anesthesiology, Critical Care and Pain Medicine, Salzburg General Hospital, Paracelsus Medical University, Salzburg, Austria; The Hospital for Sick Children and The University of Toronto, Canada

## Abstract

We previously demonstrated that intratracheally administered S-ketamine inhibits alveolar fluid clearance (AFC), whereas an intravenous (IV) bolus injection had no effect. The aim of the present study was to characterize whether continuous IV infusion of S-ketamine, yielding clinically relevant plasma concentrations, inhibits AFC and whether its effect is enhanced in acute lung injury (ALI) which might favor the appearance of IV S-ketamine at the alveolar surface. AFC was measured in fluid-instilled rat lungs. S-ketamine was administered IV over 6 h (loading dose: 20 mg/kg, followed by 20 mg/kg/h), or intratracheally by addition to the instillate (75 µg/ml). ALI was induced by IV lipopolysaccharide (LPS; 7 mg/kg). Interleukin (IL)-6 and cytokine-induced neutrophil chemoattractant (CINC)-3 were measured by ELISA in plasma and bronchoalveolar lavage fluid. Isolated rat alveolar type-II cells were exposed to S-ketamine (75 µg/ml) and/or LPS (1 mg/ml) for 6 h, and transepithelial ion transport was measured as short circuit current (ISC). AFC was 27±5% (mean±SD) over 60 min in control rats and was unaffected by IV S-ketamine. Tracheal S-ketamine reduced AFC to 18±9%. In LPS-treated rats, AFC decreased to 16±6%. This effect was not enhanced by IV S-ketamine. LPS increased IL-6 and CINC-3 in plasma and bronchoalveolar lavage fluid. In alveolar type-II cells, S-ketamine reduced ISC by 37% via a decrease in amiloride-inhibitable sodium transport. Continuous administration of IV S-ketamine does not affect rat AFC even in endotoxin-induced ALI. Tracheal application with direct exposure of alveolar epithelial cells to S-ketamine decreases AFC by inhibition of amiloride-inhibitable sodium transport.

## Introduction

Alveolar fluid clearance (AFC) is the primary mechanism for the resolution of pulmonary alveolar edema. The removal of edema fluid depends on active sodium (Na^+^) transport across the alveolar epithelial barrier. Na^+^ enters the cell via apical epithelial Na^+^-channels (ENaC) and is extruded on the basolateral side by the Na^+^/K^+^-ATPase. Chloride follows passively and alveolar fluid is reabsorbed across the epithelium driven by the resulting osmotic gradient [1], [2].

The clinical importance of maintaining consistent AFC has been documented over the last 20 years [3]–[6]. Phamacological stimulation of AFC by intravenous (IV) salbutamol reduces extravascular lung water and improves gas exchange in patients with acute lung injury (ALI) [7]. Moreover, in patients with ALI, impaired AFC is associated with shorter survival [6], [8]. Much effort has, therefore, been focused on identifying pathogenic mechanisms underlying perturbed AFC in patients with ALI, and on maintaining AFC [7], [9]–[11].

S-ketamine is frequently used for analgosedation in intensive care medicine, especially for patients with cardiac and hemodynamic instability, e.g. in sepsis [12]–[15]. We have previously demonstrated that S-ketamine decreases transalveolar Na^+^ transport and AFC in rats when it is administered into the airways but not when it is given as an IV bolus injection [16]. However, this finding does not exclude the possibility at higher plasma concentrations of S-ketamine, as regularly occurs in the clinical setting upon continuous IV analgosedation, S-ketamine crosses the endothelial-epithelial barrier to reach a concentration at the alveolar surface that is high enough to inhibit AFC. This might be especially true when the permeability of the alveolar-capillary barrier is increased, e.g. in sepsis-induced ALI.

We hypothesized that continuous IV infusion of S-ketamine for 6 hours, resulting in a considerably higher concentration of S-ketamine both in blood and in alveolar lining fluid than a single bolus, inhibits alveolar Na^+^ transport and AFC of the rat *in vivo*. Furthermore, we hypothesized that induction of a lipopolysaccharide (LPS)-induced ALI enhances the effect of continuously administered IV S-ketamine on lung fluid clearance.

## Methods

### Animals and procedures

**Figure 1:**
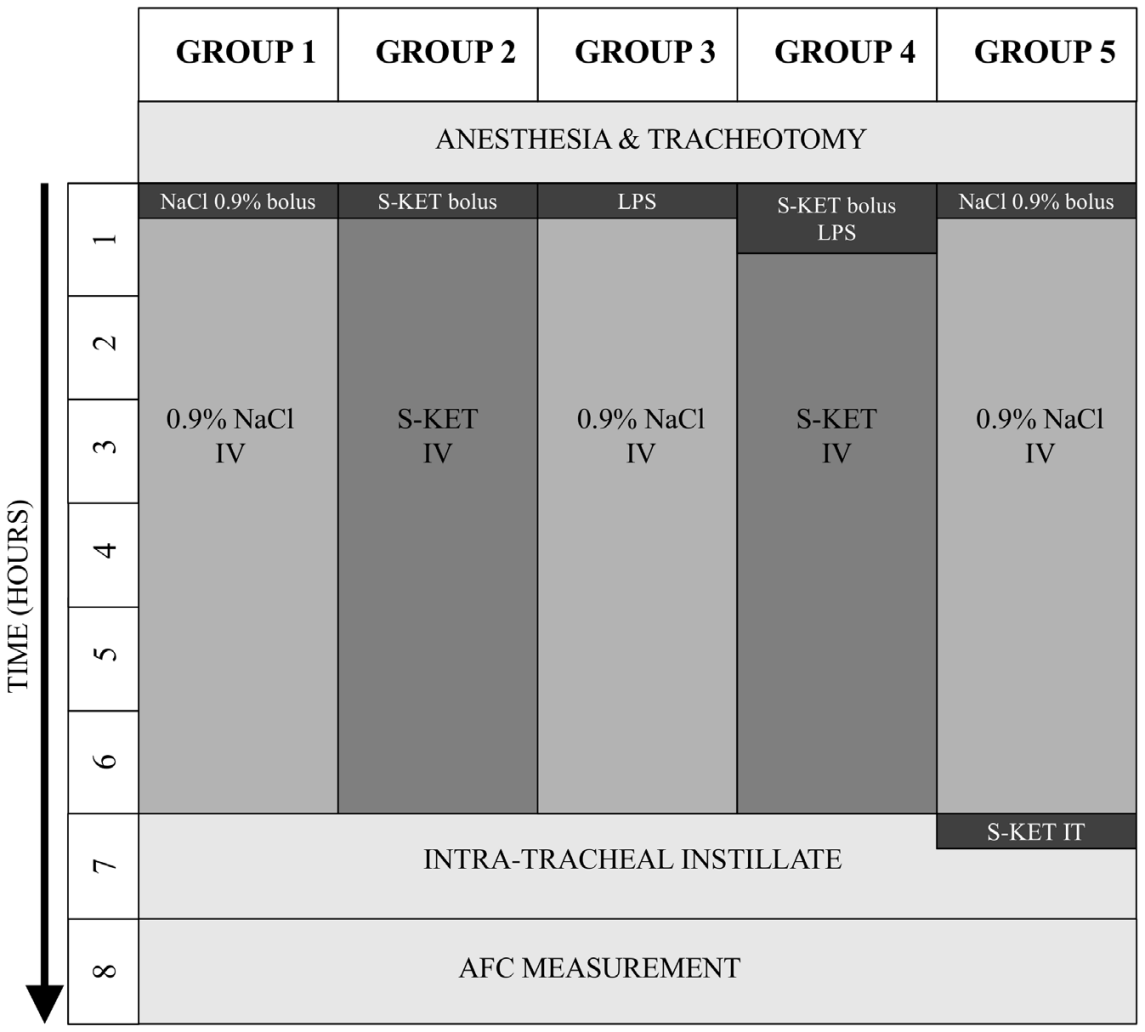
Study groups. Group 1 and Group 5 received a bolus injection of 0.9% NaCl to keep the injection stress consistent in all groups, followed by a continuous infusion of 0.9% NaCl at a rate of 7.5 ml/kg/h for 6 hours. In Group 2, an IV S-ketamine bolus of 20 mg/kg was administered followed by a continuous infusion of 20 mg/kg/h for 6 hours. Group 3 received an IV bolus injection of 7 mg/kg LPS, followed by continuous infusion of 0.9% NaCl for 6 hours. Group 4 was injected with 7 mg/kg LPS IV, and an IV loading dose of 20 mg/kg S-ketamine. This bolus injection was followed by continuous IV infusion of S-ketamine at 20 mg/kg/h for 6 hours. After 6 hours, 3 ml of a pre-warmed (37°C) instillate (3 ml) containing isothiocyanate-labeled dextran (4 µg/ml) was delivered via a tracheal cannule for measurement of AFC. In Group 5, the instillate (3 ml) was supplemented with 75 µg/ml S-ketamine (3 µl).

All experiments were approved by the Animal Protection Committee of the University of Heidelberg and the Regierungspräsidium Karlsruhe. Male Sprague-Dawley rats (Charles-River-Wiga Laboratories, Sulzfeld, Germany), weighing 302±9 g, were housed with free access to standard chow and water. Thirty animals were randomly assigned to five study groups (Figure 1). All rats were anesthetized by intraperitoneal (IP) injection of 100 mg/kg thiopental (Inresa Arzneimittel, Freiburg, Germany) as previously described [17], [18]. Tracheotomy was performed after confirming depth of anesthesia by absence of response to paw compression. Intravenous access was obtained by placing a polyethylene catheter into the femoral vein. Rats received a continuous IV infusion of either NaCl 0.9% or S-ketamine for 6 hours as indicated by the group-specific experimental protocol (Figure 1). After 6 hours, blood samples were drawn from the femoral artery and tension of carbon dioxide (pCO_2_) and oxygen (pO_2_) were measured (Rapidpoint 400/405, Siemens, Germany). To simulate pulmonary edema and subsequently quantify AFC, an instillate of 3 ml pre-warmed (37°C) isothiocyanate-labeled dextran (4 µg/ml) was delivered via a tracheal cannule as previously described [16], [19]. In rats that did not receive S-ketamine, anesthesia was maintained by repeated administration of thiopental when a response to paw compression was observed. Temperature of the rats (37.5–38.5°C) was regulated by a heating pad and continuously controlled by rectal thermometer throughout the experiment as done previously [16], [20].

### Experimental groups

Animals were randomly assigned to one of the following study groups (Figure 1):

Group 1 (Control; n = 6): Rats received a bolus injection of 0.9% NaCl to maintain consistent injection stress in all groups, followed by a continuous IV infusion of 0.9% NaCl at a rate of 7.5 ml/kg/h for 6 hours to compensate for urinary and insensible water losses. At this infusion rate, hemoglobin levels remained above 11.5 g/dl in all animals.

Group 2 (S-ketamine IV; n = 6): To study the effects of IV S-ketamine on AFC, rats received an IV loading dose of 20 mg/kg S-ketamine (Pfizer Pharma, Berlin, Germany). This bolus injection was followed by continuous IV infusion of S-ketamine at a dose of 20 mg/kg/h administered at 7.5 ml/kg/h for 6 hours.

Group 3 (LPS IV; n = 6): In order to study the effects of endotoxemia on AFC, rats received an IV bolus injection of 7 mg/kg LPS (Escherichia coli 0111:B4, Sigma-Aldrich, Deisenhofen, Germany), followed by continuous infusion of 0.9% NaCl administered at 7.5 ml/kg/h for 6 hours.

Group 4 (LPS/S-ketamine IV; n = 6): To investigate whether endotoxemia-induced lung injury alters the effect of S-ketamine on AFC, rats received an IV bolus injection of 7 mg/kg LPS, followed by an IV loading dose of 20 mg/kg S-ketamine. This bolus injection was followed by continuous IV infusion of S-ketamine at a dose of 20 mg/kg/h administered at 7.5 ml/kg/h for 6 hours.

Group 5 (S-ketamine intratracheally; n = 6): Rats received a bolus injection of 0.9% NaCl follwed by a continuous IV infusion of 0.9% NaCl at a rate of 7.5 ml/kg/h for 6 hours. To investigate the effect of S-ketamine on AFC when administered directly into the alveolar space, 75 µg/ml S-ketamine was added to the intratracheally administered instillate.

### Measurement of alveolar fluid clearance (AFC)

AFC was measured as described previously [16], [19]. Briefly, pre-warmed (37°C) instillate (3 ml) was delivered through a tracheal cannula. The instillate was composed of (in mM) 135 NaCl, 5 KCl, 1 KH_2_PO_4_, 1 MgSO_4_, 1 CaCl_2_, 5 glucose, 10 HEPES, 2% dextran-500, and fluorescein isothiocyanate-labeled dextran (4 µg/ml), pH 7.4 at 37°C, 300 mosmol/kg H_2_O. In a subset of experiments (Group 5), S-ketamine (75 µg/ml) was added to the instillate to investigate the effect of S-ketamine on AFC when administered directly into the alveolar space. Rats were not ventilated during reabsorption measurements and cardiac arrest occurred within the first minute after instillation as reported by us and others [16], [20]. It has been shown previously that cessation of blood flow and ventilation does not affect the rate of AFC over a period of 4 hours [21]. To quantify AFC in fluid instilled lungs, fluorescence, as an indicator of volume changes, was measured in aliquots of the instillate collected immediately and 60 min after fluid instillation (Labsystems Fluoroskan Ascent, Frankfurt, Germany).

### Measurement of inflammatory markers

After continuous IV infusion of either NaCl 0.9% or S-ketamine for 6 hours samples of blood plasma and bronchoalveolar lavage fluid (BALF) were obtained from groups 1–4 to determine the concentration of Interleukin (IL)-6 and cytokine-induced neutrophil chemoattractant (CINC)-3 by ELISA (R&D Systems, Abingdon, United Kingdom). The concentration of S-ketamine in plasma and BALF were measured by HPLC as described previously [16].

### Cell isolation and culture

In a different set of experiments, the effect of S-ketamine (75 µg/ml) on transalveolar ion transport was measured on primary cultured rat alveolar type II (ATII) cells as previously described [16], [22], [23]. Formation of tight monolayers was tested by measuring transepithelial resistance (epithelial voltohmmeter device and chopstick electrodes, World Precision Instruments, Sarasota, FL). In a subset of experiments, LPS (1 µg/ml) was added to the cultured cells 6 hours before transalveolar ion transport was measured.

### Ussing chamber measurements

To test whether exposure of ATII cells to 6 hours of S-ketamine affected transalveolar ion transport, short circuit currents (ISC) were measured in the Ussing chamber (n = 6 per group) as decribed previously [16]. The specific Na^+^-channel blocker amiloride (100 µM, Sigma-Aldrich, Deisenhofen, Germany) was added to the apical side of the cells to investigate which component of ISC was inhibited by S-ketamine. The amiloride-inhibitable portion of ISC reflects the degree of ion transport via epithelial Na^+^ channels (i.e. ENaC).

### S-ketamine binding to surfactant

To test whether S-ketamine binds to alveolar surfactant, increasing concentrations of S-ketamine (0, 10, 50, 100, 500 µg/ml dissolved in 540 µl Krebs buffer) were incubated for 15 min with 60 µl (4.8 mg) surfactant (Curosurf, Chiesi Pharmaceuticals, Vienna, Austria) and centrifuged (16 000×g, 5 min, RT). Thereafter, the absorption of S-ketamine in the supernatant was measured photometrically (wavelength: 270 nm; LS 500 spectral photometer, Dr. Bruno Lange, Düsseldorf, Germany).

### Statistical Analysis

Normal distribution of the data was tested using the Kolmogorov-Smirnov test. For normally distributed variables a two-way analysis-of-variance (ANOVA) was used, in which S-ketamine versus vehicle was Factor 1, and LPS versus vehicle was Factor 2. Variables not normally distributed were compared using Kruskal-Wallis analysis of variance on ranks. Pairwise multiple comparisons were made using the Student-Newman-Keuls-Method. Unpaired t-tests were used to compare two group means. Data are expressed as mean values±SD. Level of significance was p<0.05.

## Results

### Effect of S-ketamine on AFC in the fluid-instilled rat lung *in vivo*

**Figure 2:**
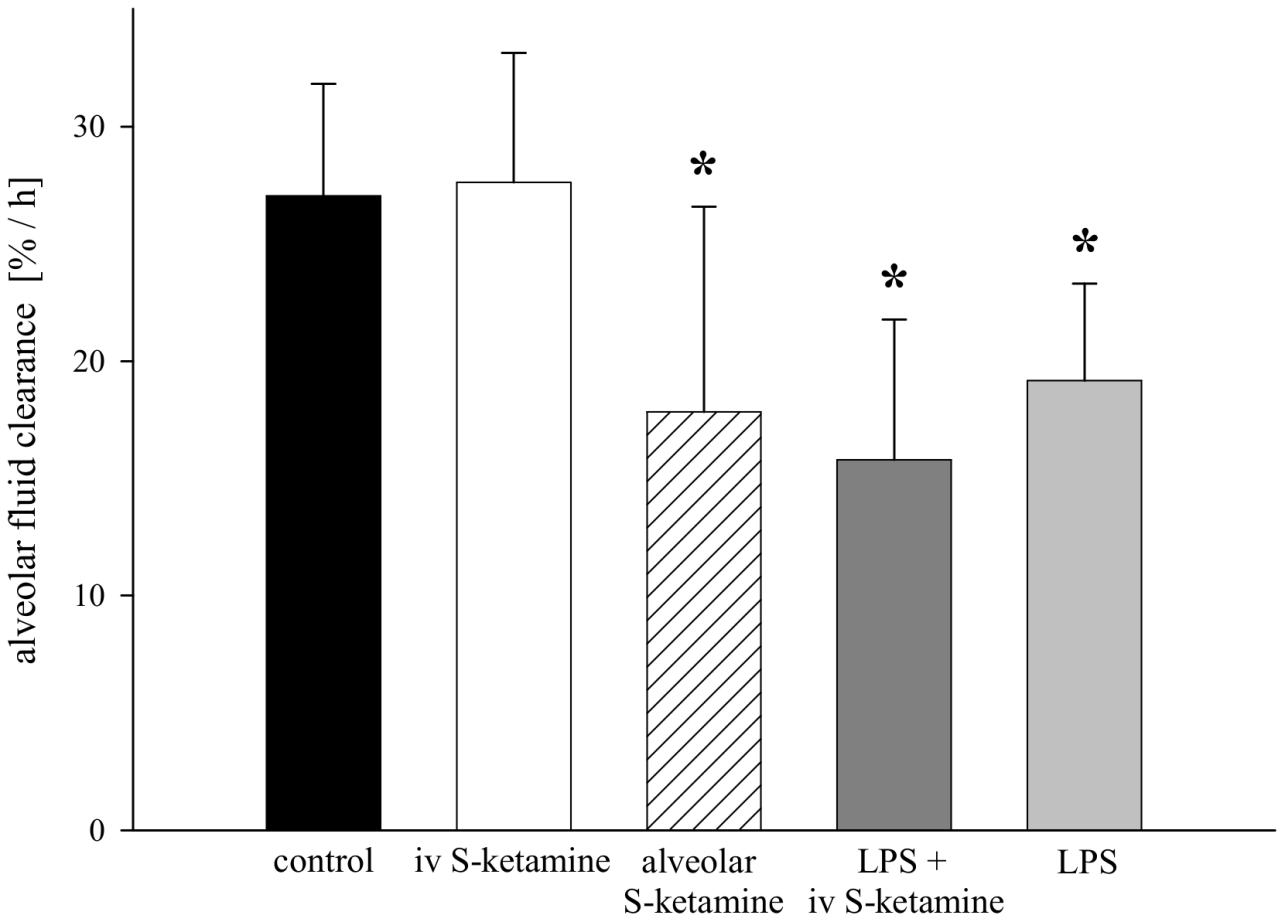
Effect of S-ketamine on alveolar fluid clearance (AFC). S-ketamine was administered IV over 6 h (loading dose: 20 mg/kg, followed by 20 mg/kg/h) or administered directly into the alveolar space by addition to the instillate (75 µg/ml). Mean values±SD of 6 experiments per group. *Significant difference (p<0.05) versus control and versus IV S-ketamine.

As summarized in Figure 2, AFC of control rats was 27±5% over 60 min. Intravenous S-ketamine did not affect AFC (p = 0.44 versus control). In contrast, IV LPS decreased AFC to about 16±6% (p<0.001). The combination of IV S-ketamine with LPS had no additional effect compared to LPS alone. However, when S-ketamine was administered to the alveolar side by addition to the instillate, AFC of control rats decreased to 18±9% (p = 0.01 versus control).

### Effect of LPS on inflammatory mediators in plasma and bronchoalveolar lavage fluid

These measurements were made to verify that LPS treatment induced systemic and pulmonary inflammation. As summarized in Table 1, LPS increased the concentration of IL-6 and CINC-3 in plasma and bronchoalveolar lavage fluid after 6 hours (both p<0.01), indicating a significant inflammatory response. Treatment with S-ketamine did not affect IL-6 or CINC-3 levels (Table 1).

**Table 1:** Changes in markers of inflammation in rat plasma and bronchoalveolar lavage fluid upon LPS application.

	Controls	IV S-ketamine	LPS	IV S-ketamine+LPS
IL-6 in plasma [pg/ml]	<50.76	<50.76	10896±7645*	8065±1404*
IL-6 in BALF [pg/ml]	549±431	272±157	4685±2788*	4147±1336*
CINC-3 in plasma [pg/ml]	<50.76	<50.76	519±155*	731±394*
CINC-3 in BALF [pg/ml]	276±196	92±14	5032±1715*	5107±1800*

Mean values±SD of 6 experiments per group, 6 hours after IV injection of lipopolysaccharide (LPS; 7 mg/kg). CINC-3: Cytokine-induced neutrophil chemoattractant 3; IL-6: Interleukin 6. (*) indicates significant difference (p<0.01) versus control.

### Effect of LPS on S-ketamine concentrations in plasma and bronchoalveolar lavage fluid

Administration of IV S-ketamine resulted in a plasma concentration of 8.81 µg/ml at the end of the 6-hour treatment (Table 2). The concomitant S-ketamine concentration in BALF was about 8% of its plasma concentration. In rats with LPS-induced endotoxemia, IV administration of S-ketamine yielded a twofold higher plasma concentration compared to the plasma concentration in healthy rats (p<0.05; Table 2). However, the concomitant S-ketamine concentration in BALF was not increased. When S-ketamine (75 µg/ml) was administered intratracheally, its concentration in BALF was about twice as high as after IV injection (p<0.05; Table 2).

**Table 2:** S-ketamine concentrations in plasma and bronchoalveolar lavage fluid.

	IV S-ketamine	IV S-ketamine+LPS	Intratracheal S-ketamine
S-ketamine concentration			
- in plasma [µg/ml]	8.81±7.29	17.84±10.98*	0.04±0.03*#
- in BALF [µg/ml]	0.70±0.40	0.74±0.54	1.57±0.53*#

Mean values ± SD of 6 experiments per group, 6 hours after injection of IV S-ketamine (loading dose: 20 mg/kg, followed by 20 mg/kg/h), of IV S-ketamine in combination with IV lipopolysaccharide (LPS; 7 mg/kg), and tracheal S-ketamine [75 µg/ml] (*) indicates significant difference (P<0.05) versus IV S-ketamine.

# indicates significant difference (p<0.05) versus IV S-ketamine+LPS.

### Effect of S-ketamine on ion transport of primary cultured rat ATII cells

**Figure 3:**
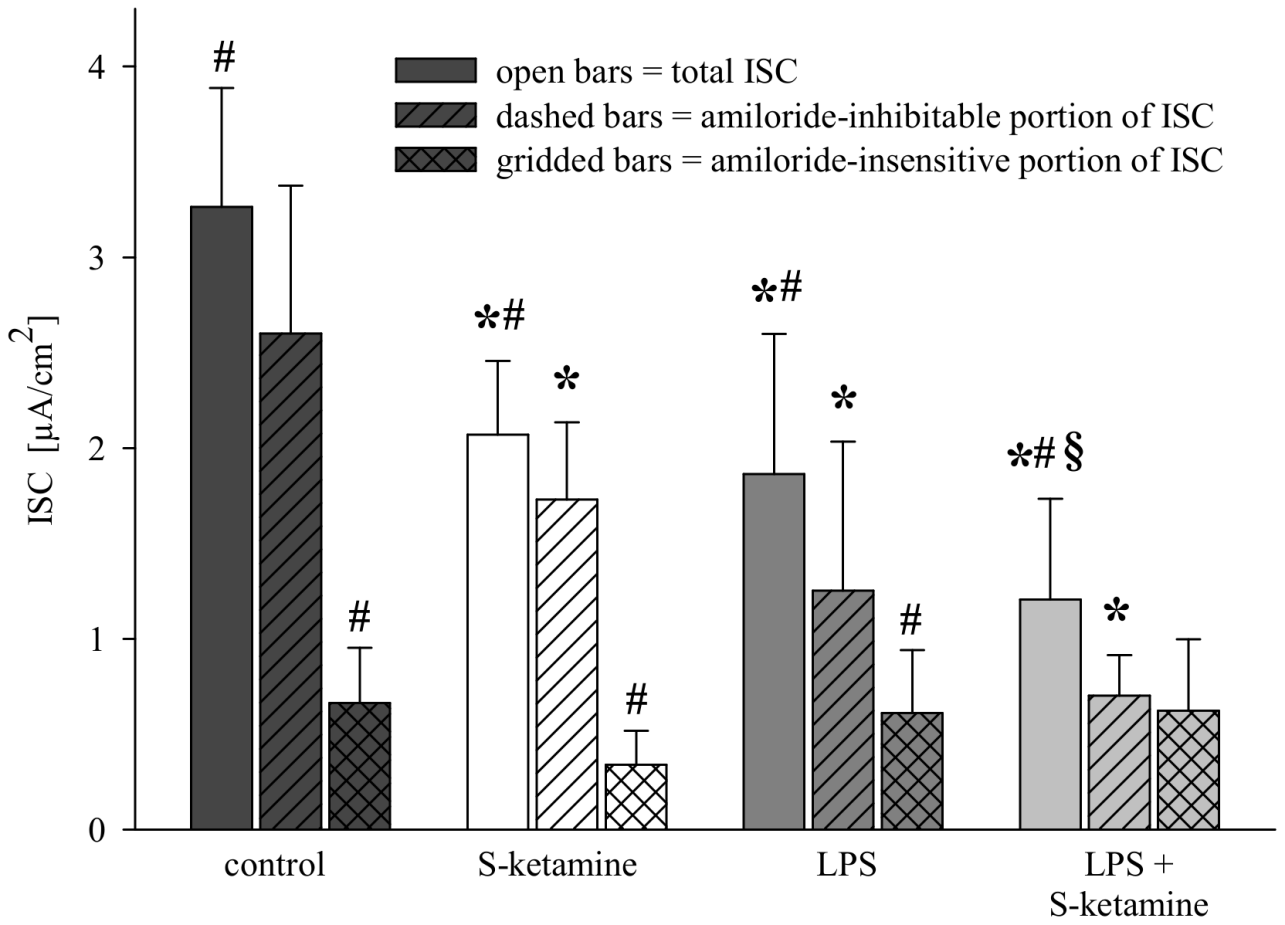
Short circuit currents (ISCs) of primary cultured rat alveolar type II (ATII) cells after 6 hours' incubation with S-ketamine (75 µg/ml) or lipopolysaccharide (LPS; 1 µg/ml). Some experiments (dashed bars) were performed with the specific Na^+^-channel blocker amiloride (100 µM) to determine which component of ISC was inhibited by S-ketamine. The amiloride-inhibitable portion of ISC reflects the degree of ion transport via ENaC. Open bars indicate total ISC, dashed bars show the amiloride-inhibitable portion of ISC (Δ amiloride), and cross-hatched bars the amiloride-insensitive portion of ISC. For example, in control cells total ISC was 3.3±0.6 µA/cm^2^. Of this current, 2.6±0.8 µA/cm^2^ were caused by amiloride-inhitable Na^+^-channels (i.e. the ENaC), and 0.7±0.1 µA/cm^2^ were caused by amiloride-insensitive ion channels. Mean values±SD of 6 experiments per group. *Significant difference (p<0.01) versus control. #Significant difference (p<0.05) versus amiloride-inhibitable portion of the same treatment condition. $Significant difference (P<0.05) versus S-ketamine.

ISC of cultured primary alveolar epithelial cells was measured to verify that direct pre-exposure of ATII cells to S-ketamine for 6 hours affects transepithelial transport. In untreated control cells ISC was 3.3±0.6 µA/cm^2^ (open bar; Figure 3). Pre-exposure to S-ketamine for 6 hours decreased ISC by about 37% to 2.1±0.4 µA/cm^2^ (p<0.01; Figure 3), reflecting inhibition of transalveolar ion movement. Pre-treatment with LPS for 6 hours decreased ISC by about the same magnitude (p<0.01 versus control). The combination of S-ketamine with LPS caused stronger inhibition than either substance alone and decreased ISC by about 63% (p<0.01; Figure 3).

The Na^+^ channel blocker amiloride was added to the cells to characterize the component of ISC that was inhibted by S-ketamine. In cells that had been pre-treated with S-ketamine, the amiloride-inhibitable portion of ISC (dashed bars, Figure 3) was significantly smaller (1.7±0.4 µA/cm^2^) than in untreated control cells (2.6±0.8 µA/cm^2^; p<0.05; Figure 3), indicating that S-ketamine had partially inhibited ENaC. In line with this observation this reduction (−35%) was of the same magnitude as the degree of inhibition (−37%) caused by S-ketamine alone.

In cells pre-treated with LPS, the amiloride-inhibitable portion of ISC was reduced by about 50% compared to control cells (p = 0.001; Figure 3), indicating that LPS inhibited ENaC, too. In cells treated with S-ketamine and LPS, the amiloride-inhibitable portion was further reduced (0.7±0.2 µA/cm^2^; p<0.001 versus control; Figure 3).

The amiloride-insensitive portion of ISC (cross-hatched bars, Figure 3), which is generated by ion channels other than the ENaC, was not different between the experimental groups (P = 0.44). This result indicates no inhibition from either S-ketamine or LPS inhibited this portion.

### Binding of S-ketamine to surfactant

**Figure 4:**
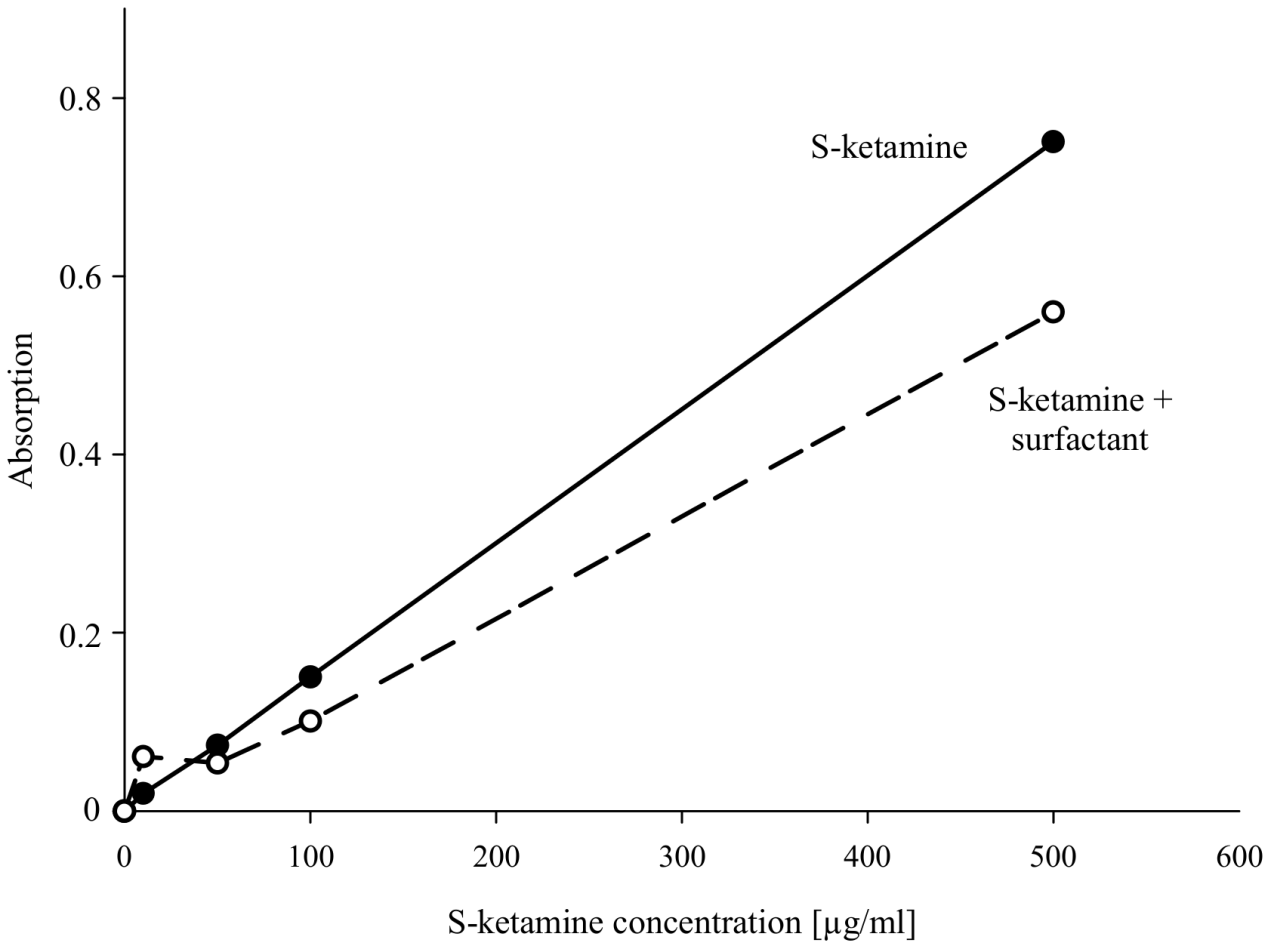
Absorption measurements of increasing concentrations of S-ketamine (0, 10, 50, 100, 500 µg/ml dissolved in Krebs buffer) without (filled symbols) or after (open symbols) 15 min incubation with surfactant. After centrifugation (16000×g, 5 min), absorption was measured photometrically (wavelength: 270 nm) in the supernatant.

Increasing concentrations of S-ketamine (0–500 µg/ml) caused a linear increase in photometric absorption when dissolved in Krebs buffer (Figure 4, filled symbols). In contrast, in the presence of surfactant, only S-ketamine concentrations >50 µg/ml increased absorption. Under these conditions the dose-response curve was shifted to the right (Figure 4, open symbols), indicating binding of S-ketamine to surfactant.

### Effect of S-ketamine and LPS injection on blood gases

The effects of S-ketamine and LPS injection on blood gases at 6 hours are shown in Table 3. There were no significant differences in arterial pCO_2_ and pO_2_ between the different treatment groups.

**Table 3:** Effect of S-ketamine and LPS on blood gases.

	Controls	IV S-ketamine	LPS	IV S-ketamine+LPS
PCO_2_ [torr]	34.5±4.7	35.7±3.3	30.7±3.7	31.6±3.7
PO_2_ [torr]	79.0±15.6	65.6±15.4	67.9±6.9	62.7±18.3

Mean values ± SD of 6 experiments per group at 6 hours after injection of IV S-ketamine (loading dose: 20 mg/kg, followed by 20 mg/kg/h), of IV lipopolysaccharide (LPS; 7 mg/kg), and of IV S-ketamine in combination with IV LPS, respectively.

## Discussion

We have previously demonstrated that administration of S-ketamine into the rat's alveolar space inhibits AFC, whereas an IV bolus injection does not [16]. However, in the clinical setting S-ketamine is typically used for IV analgosedation. We, therefore, hypothesized that during continuous IV infusion, S-ketamine reaches clinically relevant plasma concentrations, which, after crossing the endothelial-epithelial barrier, yield a concentration at the alveolar surface high enough to inhibit AFC. We thought that inhibition might even be aggravated when the permeability of the alveolar-capillary barrier is increased, e.g. in sepsis-induced ALI. However, disproving our hypothesis, the present study shows that 6 hours' IV infusion of S-ketamine does not affect AFC in healthy rats or in rats with endotoxemia-induced ALI.

AFC was measured in fluid-instilled rat lungs, which is a well-established model for studying lung fluid balance *in vivo*. Control rats cleared about 27% of the instilled fluid over 60 min, which is in line with other studies [24], [25]. Against our hypothesis AFC was not affected in rats treated with IV S-ketamine for 6 hours. We further investigated whether IV S-ketamine affects AFC when the permeability of the alveolar-capillary barrier is increased. However, even upon LPS-induced endotoxemia and ALI S-ketamine did not affect lung fluid balance.

We induced only mild ALI to preserve the integrity of the alveolar epithelium, which is a prerequisite for maintaining AFC [8]. A more severe lung injury is associated with alveolar flooding, a marked increase in epithelial permeability to protein, and an inability to transport fluid from the air spaces of the lung [5]. It is conceivable that in this situation S-ketamine might inhibit AFC. However, when the alveolar epithelium is disrupted, drug-induced inhibition of AFC is of limited clinical significance. In LPS treated rats about 16% of the instilled fluid was still reabsorbed, indicating that the function of the alveolar epithelium was sufficiently preserved to allow net clearance of alveolar fluid despite significant pulmonary inflammation. The latter was indicated by elevated concentrations of the inflammatory parameters CINC-3 and IL-6 in plasma and BALF, both of which play key roles in the pathophysiology of ALI [26], [27].

We can only speculate as to the reasons that IV S-ketamine did not inhibit AFC. One possibility might be an anti-inflammatory effect of S-ketamine [28], but our measurements of the inflammatory parameters in plasma and BALF do not support this notion. It also appeared possible that the 6 hours' exposure rendered the alveolar epithelium insensitive to S-ketamine. However, our Ussing chamber experiments demonstrate that incubation of ATII cells with S-ketamine for 6 hours decreased transalveolar Na^+^-transport, indicating that this exposure time does not render transalveolar ion transport insensitive to S-ketamine. The results further show that in S-ketamine treated cells, the amiloride-inhibitable portion of ISC was significantly reduced. This finding suggests, that S-ketamine partially inhibited ENaC, which might explain the inhibition of reabsorption upon alveolar application, which is in line with previous observations [16], [29].

The question arises whether the alveolar concentration of S-ketamine after IV administration was high enough to inhibit epithelial Na^+^-transport. However, the concentration we used for IV infusion of S-ketamine elicited sufficient depth of anesthesia and yielded plasma concentrations that were considerably higher than the 2–3 µg/ml that are usually targeted for IV analgosedation with S-ketamine in humans [30]–[32]. Thus, the dosage and plasma concentration of S-ketamine were high enough to be of clinical and pharmacological relevance. Even at these relatively high plasma concentrations, IV S-ketamine did not affect AFC, suggesting that the lower plasma levels that usually occur in the clinical setting also do not impair AFC.

After IV administration, the S-ketamine concentration in BALF was about 0.7 µg/ml in control rats, demonstrating that IV S-ketamine entered the alveolar space even when the alveolar-capillary barrier was intact. Induction of an endotoxin-induced ALI did not further increase the S-ketamine concentration in BALF. This observation suggests that increased leakiness of the endothelial-epithelial barrier induced by inflammation does not affect the distribution of S-ketamine, which can be explained by the relatively free diffusibility of S-ketamine associated with its lipophilic structure.

Tracheal application of S-ketamine at a concentration of 75 µg/ml reduced AFC to 18% over 60 min. This indicates that the S-ketamine concentration was high enough to bind to Na^+^ channels located at the apical surface of alveolar epithelial cells. Immediately after instillation the S-ketamine concentration in BALF was about 1.5 µg/ml. This decrease in concentration (from 75 to 1.5 µg/ml) indicates that S-ketamine disappeared from the alveolar space, which might be explained by rapid distribution of S-ketamine via the circulation into other body compartments. Another possibility is binding of the lipophilic S-ketamine to alveolar surfactant since the major components of pulmonary surfactant include phospholipids, neutral lipids and the surfactant proteins B and C [33]. Indeed, our *in vitro* experiments showed that up to a concentration of 50 µg/ml S-ketamine was completely bound by surfactant, whereas at higher concentrations, binding by surfactant appeared to be saturated. While the initial peak concentration of S-ketamine (75 µg/ml) after tracheal application did exceed the binding capacity of surfactant, the peak concentration in the alveolar space after IV injection did not. The S-ketamine concentration we measured in BALF after IV injection was about 0.7 µg/ml, which is below the binding capacity of surfactant. These findings suggest that S-ketamine, when administered into the alveolar space at a concentration that exceeds the binding capacity of surfactant, inhibits alveolar Na^+^ transport and decreases AFC by inhibition of amiloride-inhibitable epithelial Na^+^ channels. However, in severe ALI and ARDS, surfactant secretion might be decreased, and surfactant might be removed from the alveolar surface by alveolar edema [34]. It is conceivable that in this situation, S-ketamine can not be neutralized by the alveolar epithelial surfactant film and that it then exerts its full capability of inhibition of alveolar reabsorption.

### Limitations

One limitation of this study is that we did not establish a dose response curve for the effect of alveolar S-ketamine on AFC. However, due to the data variation typical of AFC measurements, these studies would have required a large number of rats. Therefore, only one time point, and only one IV dosage were investigated. The S-ketamine concentration used for intratracheal application was chosen in order to approximately match both the S-ketamine concentration in the BALF after an IV injection and after intra-tracheal administration.

To investigate whether S-ketamine is bound by surfactant, we had to use porcine lung surfactant, because rat lung surfactant was not available. The discordant results on transport inhibition on ATII cell monolayers and lack of inhibition upon IV application are difficult to explain. They might have to do with altered surfactant production in cultured monolayers [35].

Due to the many between-species variations in the properties of the ENaC, our findings do not exclude the possibility that IV S-ketamine might reduce ENaC activity – and thus AFC – in humans, but not in rats, as suggestedpreviously [29], [36].

### Perspectives

The present study focused on the effects of IV S-ketamine on AFC in a rat model of LPS-induced endotoxemia and mild ALI. Further animal studies may target at the role of IV S-ketamine in more severe lung injury and in situations with dysfunction or decreased secretion of surfactant. Due to the limitations of animal models in terms of their their relevance in human systems the present findings should be interpreted with caution when being extrapolated to the human situation.

The first studies documenting that S-ketamine in the rat alveolar space impairs AFC [16], [29] raised the question whether S-ketamine might be disadvantegous as analgosedative drug in patients with ALI. The present study does not support this concern. However, future studies targeting pathogenic mechanisms underlying perturbed AFC in patients with ALI are necessary to evaluate the safety of IV S-ketamine in this situation.

## Conclusions

Our study shows that continuous IV infusion of S-ketamine at clinically relevant concentrations for 6 hours does not impair alveolar fluid reabsorption in healthy rats or in rats with mild endotoxemia-induced ALI, whereas alveolar S-ketamine inhibits reabsorption. It appears that surfactant has a certain capacity to bind S-ketamine, and that an intact layer of surfactant prevents inhibition of reabsorption, even in mild ALI.
